# Tuberculosis in Private Practice

**Published:** 1919-08-30

**Authors:** 


### TUBERCULOSIS IN PRIVATE PRACTICE.

### Some Points in Early Diagnosis and Prevention.

With each passing year there is undoubtedly an increasing tendency to specialism in medicine, and although this gradual change has, in numberless instances, proved far from beneficial to either the interest or income of the general practitioner, yet it was inevitable and, we believe, promises in the long run to produce more perfect control and treatment of disease. It is not our purpose here to suggest the ideal professional behaviour of either the specialist or the general worker in furthering the close co-ordination required in tuberculosis matters. The local man has but to realise that experience necessarily enables the consultant not only to be more accurate in excluding the disease when absent, and in detecting its earliest signs, but also in sympathising with the difficulties of diagnosis and more particularly of treatment under the average conditions of private practice. In this, as in all branches of the profession, it is the obvious duty of the specialist to be scrupulously careful to ensure that his colleague receives all possible credit out of the case, and to avoid the slightest action which may lessen the patient's confidence in his own doctor, and these elementary facts thus briefly impressed, we may pass on to some considerations in the actual control of tubercle infection as met with in general practice.

### The Tragedies of the Disease.

It is a regrettable fact that many instances occur where the tragedies of this disease follow upon weeks or months of delay while waiting, in absence of obvious bacteriological evidence, for classical signs to develop. There is a natural hesitation to risk the making of a prematurely positive diagnosis when the verdict represents almost life itself to the mind of the average patient. But at the same time we realise that the process of medical education is essentially gradual. We were taught the typical stages of "phthisis" with early symptoms of cough, expectoration, loss of weight, and hæmoptysis, but it is nowadays fully realised that not one of these is associated with the earliest invasion of the lungs by tubercle. By the time we can read the diagnosis in the patient's face, and we begin our search for acid-fast bacilli in his sputum, he has infected perhaps dozens of his fellows and is himself possibly far beyond cure. How many patients are rushed off to sanatoria at this stage, when the clinical picture is obvious and typical? To the care of specialists they go then, but how often is it too late? The skilled attention and expense lavished upon them produce a certain percentage of cures, and one appreciates the value of the institutions and the ability of the men to whom they are handed over, but how greatly could we reduce the incidence of such advanced cases if there were more general realisation of the possibility of earlier diagnosis?

Dr. Sutherland raised many of the finer points in connection with work in private practice when delivering his recent address before the Tuberculosis Society, and coming from a man of such long experience, the summary of early signs which he then gave are well worth noting.

Lung trouble is probably unmentioned by the patient. Nervous irritability increasing as the day advances, lassitude, constipation, loss of appetite, amenorrhœa, are all early symptoms. Fever, loss of weight, cough, breathing difficulties, and typical sputum follow much later, but none are pathognomonic of pulmonary tuberculosis. Even nightsweats, emaciation, or hæmoptysis may be symptoms arising from quite other causes, and each must be considered in relation to actual physical signs. Those mentioned include microadenopathy above the clavicles, dystrophy of the trapezius muscles with sharpened contour of the shoulders, diminished tidal expansion and rapid tailing off in resonance at the apex, narrowing of the shoulder area of resonance, with an alteration of the respiratory murmur, and strongly suggest a tuberculous process when symptoms are also present. If, however, such conditions prevail that means do not exist for complete physical examination for either excluding tuberculosis of finding another cause for the symptoms, then we would urge that the patient be sent either to a tuberculosis officer or a private specialist. It is in the interest of the community.

### Tuberculin Reactions.

There is also a diagnostic test of which more use might be made—namely, Von Pirquet's cutaneous tuberculin reaction. By no means infallible and not even meeting with general acceptance, this test, when definitely negative or strongly positive, can, and does in our own experience, give most useful information. The *sub*cutaneous tests are, however, to be considered distinctly dangerous.

Among the most important points which the general practitioner should always remember, comes the fact that in nearly every household which contains or has contained an infectious tuberculous patient, there will be other cases of the disease, early though they may be, awaiting recognition. Also that in these early cases, if they be diagnosed in time, there is probably a 90 per cent. chance of permanent cure. The important factor lies in early diagnosis, and whether the aid of the specialist is sought or not, care of the patient may well remain in the hands of the practitioner, for such cases need not necessarily receive institutional treatment. Hundreds of such early infections, in both children and adults, are successfully cured in their own homes by free co-operation between the local doctor and an expert consultant, and one would like to see this principle more often and more practically applied to those intermediate masses which do not customarily meet the specialist either in Harley Street or as his hospital out-patients.

